# Being positive on disability in medical education

**DOI:** 10.1192/bjb.2021.48

**Published:** 2021-06

**Authors:** Elliott Carthy

**Affiliations:** University of Oxford, UK. Email: elliott.carthy@psych.ox.ac.uk

The world is a diverse place. The patients we treat are a diverse group. The same can't be said for the medical profession that represents them. I would argue that positivity regarding disabilities in medicine starts with us as educators. My favourite aspect of being a medical educator is what I as a teacher learn from my students. I, like most doctors and medical students, do not have a disability. However, I communicate with and advocate for people with intellectual disabilities, autism and learning difficulties such as dyspraxia and teach about these topics as routine practice to students and the wider multidisciplinary team. This is not just about reducing discrimination and promoting equality but also about appreciating the value that such people add to society.

General community estimates within the UK suggest the prevalence of disability in working age adults to be 19% in the UK,^1^ yet in medical schools, it is estimated that only 4.1% of students have a disability.^2^ Even accounting for potential non-disclosure, these numbers show substantial underrepresentation. The British Medical Association recently published a report titled *Disability in the Medical Profession*^3^ that highlighted the paucity of doctors and medical students with disabilities who felt safe to disclose their condition or felt supported by their institution or colleagues. Despite widening participation programmes, medicine can still be discriminatory towards those with disabilities. It would not be unreasonable to suppose that such reluctance to seek support or have the need for this recognised could be extrapolated to our patients.

The term disability is broad, both in nature and degree. This is often poorly reflected in medical guidelines, whereby our lack of understanding can contribute to the pre-existing stigma. The title of the General Medical Council's *Welcomed and Valued*^4^ guidance for those with disabilities in the medical profession gives the impression of framing inclusivity in a positive light. However, the persistent use of terms like ‘support’, ‘student needs’ or educators and institutions applying their ‘duties’ means the focus is on reducing discrimination and making reasonable adjustments to allow for an assessment of competence. The presumption is that disability may be a threat to competence and to patient safety. In fact, disability may give lived experience that improves competency through empathy and understanding. We need to start focusing on what such students, doctors, educators and, crucially, patients can add, not simply what they need.

If we provide a safe space for those with lived experience of disabilities to share their experiences and the impact on their life, then each one of us can improve our understanding. Indeed, more doctors being open about their visible and invisible disabilities mean more positive role models for students. Widening participation can then move from just implementing anti-discrimination legislation to appreciating the value that diversity adds and the grassroots barriers to entering and thriving in the profession of medicine. Every medical educator can contribute by providing a safe space for disclosure and a willingness to learn and be educated by our patients and students.

## References

[1] Department of Work & Pensions. *Family Resources Survey 2018/19*. 2020. Available from: https://www.gov.uk/government/statistics/family-resources-survey-financial-year-201819 [cited 27 Dec 2020].

[2] Shrewsbury D. Disability and participation in the professions: examples from higher and medical education. Disabil Soc 2015; 30(1): 87–100.

[3] British Medical Association. *Disability in the Medical Profession*. Available from: https://www.bma.org.uk/advice-and-support/nhs-delivery-and-workforce/workforce/disability-in-the-medical-profession [cited 27 Dec 2020].

[4] General Medical Council. *Welcomed and Valued: Supporting Disabled Learners in Medical Education and Training*. Available from: https://www.gmc-uk.org/education/standards-guidance-and-curricula/guidance/welcomed-and-valued [cited 27 Dec 2020].

